# *BRCA* Genetic Test and Risk-Reducing Salpingo-Oophorectomy for Hereditary Breast and Ovarian Cancer: State-of-the-Art

**DOI:** 10.3390/cancers13112562

**Published:** 2021-05-23

**Authors:** Masayuki Sekine, Koji Nishino, Takayuki Enomoto

**Affiliations:** Department of Obstetrics and Gynecology, Niigata University Graduate School of Medical and Dental Sciences, Niigata 951-8510, Japan; knishino@med.niigata-u.ac.jp (K.N.); enomoto@med.niigata-u.ac.jp (T.E.)

**Keywords:** *BRCA1/2*, hereditary breast and ovarian cancer, homologous recombination deficiency, risk-reducing salpingo-oophorectomy, PARP inhibitor, companion diagnosis

## Abstract

**Simple Summary:**

The *BRCA* genetic test and HRD (homologous recombination deficiency) test are used as a companion diagnosis before starting PARP (poly ADP-ribose polymerase) inhibitor treatment. In clinical practice, gynecologists treating ovarian cancer are faced with decisions such as whether to recommend germline BRCA (g*BRCA*) test to all ovarian cancer patients and whether to do the g*BRCA* test first or HRD test first. Regarding the judgment result of the HRD test, the cutoff value differs depending on the clinical trial, and the prevalence of g*BRCA* pathogenic variant rate is different in each histological type and country. RRSO (risk reducing salpingo-oophorectomy) may have possibly reduced the risk of breast cancer for BRCA2 pathogenic variant carriers but not for *BRCA*1 carriers.

**Abstract:**

In the field of gynecology, the approval of the PARP inhibitors (PARPi) has been changing the treatment of ovarian cancer patients. The *BRCA* genetic test and the HRD test are being used as a companion diagnosis before starting PARPi treatment. BRACAnalysis CDx^®^ and Myriad myChoice^®^ HRD test are widely used as a BRCA genetic test and HRD test, respectively. In addition, FoundationOne^®^CDx is sometimes used as a tumor BRCA test and HRD test. In clinical practice, gynecologists treating ovarian cancer are faced with making decisions such as whether to recommend the g*BRCA* test to all ovarian cancer patients, whether to perform the g*BRCA* test first or HRD test first, and so on. Regarding the judgment result of the HRD test, the cutoff value differs depending on the clinical trial, and the prevalence of g*BRCA* pathogenic variant rate is different in each histological type and country. A prospective cohort study showed that RRSO reduced all-cause mortality in both pre- and postmenopausal women; however, RRSO significantly reduced the risk of breast cancer for *BRCA*2 pathogenic variant carriers, but not for *BRCA*1 pathogenic variant carriers. Moreover, salpingectomy alone is said to not decrease the risk of developing ovarian or breast cancer, so further discussion is evidently required. We discuss the current situation and problems in doing *BRCA* genetic test and RRSO in this review article.

## 1. Introduction

Hereditary breast and ovarian cancer (HBOC) caused by germline *BRCA1/2* gene pathogenic variant (g*BRCA*m) is predicted to be responsible for about 5% of all breast cancers and 15% of all ovarian cancers [1,2]. The use of Poly (ADP-ribose) polymerase inhibitors (PARPi) has begun to spread in clinical practice for patients with breast cancer, ovarian cancer, and prostate cancer.

The *BRCA* genetic test and HRD test are currently used as a companion diagnosis before starting PARPi treatment. BRACAnalysis CDx^®^ (Myriad Genetic Laboratories, Salt Lake City, UT, USA) and Myriad myChoice^®^ HRD (Myriad Genetic Laboratories, Salt Lake City, UT, USA) test are widely used as a BRCA genetic test and homologous recombination deficiency (HRD) test, respectively. In addition, FoundationOne^®^CDx (Foundation Medicine, Inc.,Cambridge, MA, USA) is sometimes used as a tumor BRCA test and HRD test. On the other hand, *BRCA* genetic testing, which is performed with the family history and past medical history considered, has become widely performed in patients with suspected hereditary ovarian cancer. As a result, a large number of families harboring *BRCA* pathogenic variants were found, and surveillance and risk-reducing surgery for *BRCA* pathogenic variant carriers in the family have become important issues. The effectiveness of risk reducing salpingo-oophorectomy (RRSO) in reducing the risk of ovarian and fallopian tube cancer in *BRCA* pathogenic variant carriers has been demonstrated in a number of studies. A meta-analysis of the breast/ovarian cancer risk-reducing effect of RRSO showed that ovarian cancer risk was reduced by nearly 80% and breast cancer risk by 50%. Furthermore, a prospective cohort study showed that RRSO reduced all-cause mortality in both pre- and postmenopausal women. We discuss the current situation and problems in doing *BRCA* genetic testing and RRSO in this review article.

## 2. Genetic Testing for *BRCA* Pathogenic Variant in Ovarian Cancer Patients

### 2.1. Indication of PARP Inhibitors

Olaparib is approved for use in maintenance treatment of platinum-sensitive recurrent ovarian cancer and first-line maintenance treatment of *BRCA*-mutated and/or HRD positive advanced ovarian cancer. Based on the results of Study 19 (phase II) and the SOLO 2 (phase III) trial [3,4,5,6], olaparib is being used for the maintenance treatment of patients with recurrent epithelial ovarian, fallopian tube, or primary peritoneal cancer who are in complete or partial response to platinum-based chemotherapy, the so-called platinum-sensitive recurrent cancer. Next, based on the results of SOLO 1 (phase III) [7], olaparib was approved for the maintenance treatment of patients with deleterious or suspected deleterious germline or somatic *BRCA*-mutated advanced epithelial ovarian, fallopian tube, or primary peritoneal cancer who are in complete or partial response to first-line platinum-based chemotherapy.

Recently, the indication of olaparib was expanded to include its combination with bevacizumab for first-line maintenance treatment of patients with advanced epithelial ovarian, fallopian tube, or primary peritoneal cancer who are in complete or partial response to first-line platinum-based chemotherapy and whose cancer is associated with homologous recombination deficiency positive status defined by either a deleterious or suspected deleterious *BRCA* variant, and/or genomic instability based on the results of PAOLA-1 trial [8]. Furthermore, Myriad myChoice^®^ HRD test was also approved as a companion diagnostic for olaparib to determine the HRD and tumor ***BRCA*** variant status of the patients.

A second PARPi, niraparib, was approved for use in maintenance treatment of platinum-sensitive recurrent patients and first-line maintenance treatment of all-comer patients with advanced epithelial ovarian, fallopian tube, or primary peritoneal cancer who are in a complete or partial response to first-line platinum-based chemotherapy based on the results of ENGOT-OV16*/*NOVA and PRIMA trial [9,10]. In addition, niraparib was approved for patients with advanced ovarian, fallopian tube, or primary peritoneal cancer treated with three or more prior chemotherapy regimens and whose cancer is associated with HRD-positive status. HRD is defined by either a deleterious or suspected deleterious *BRCA* variant, or genomic instability in patients with a disease progression greater than six months after their response to the last platinum-based chemotherapy.

### 2.2. Positive Rate of gBRCA m in Patients with Epithelial Ovarian Cancer

After studying 1915 U.S. cases of epithelial ovarian cancer (including fallopian tube and peritoneal cancer), Norquist et al. reported the following frequencies of germline pathogenic variants: *BRCA* 1 and *BRCA* 2, 15% (*BRCA* 1: 8.5%, *BRCA* 2: 6.3%); high-grade serous carcinoma (HGSC), 16%; low-grade serous carcinoma (LGSC), 6%; endometrioid carcinoma, 9%; clear cell carcinoma, 7%; and mucinous carcinoma, no pathogenic variants [2]. According to this study, the frequency of *BRCA* pathogenic variants in clear cell carcinomas was unexpectedly high, implying that *BRCA* pathogenic variant-positive cancer does not necessarily lead to HGSC.

**Figure 1:**
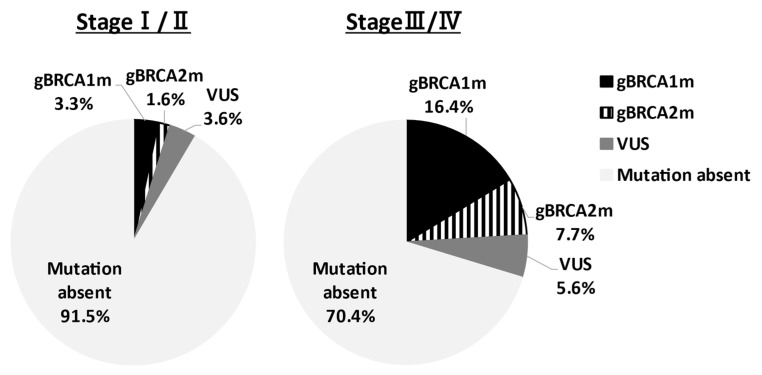
Prevalence of germline *BRCA*1/2 pathogenic variants by FIGO stage. The prevalence of g*BRCA*m of ovarian cancer patients in the Japan CHARLOTTE study was shown. The *BRCA* pathogenic variant rate was 4.9% and 24.1% in stages I–II and stages III–IV, respectively. VUS: variant of uncertain significance.

In Asia, Sekine et al. reported that HGSC accounts for 80% of ovarian cancers with g*BRCA*m [11]. Sakamoto et al. conducted *BRCA* genetic tests on 95 ovarian cancer patients and reported g*BRCA*m in 12 patients (12.6%) (*BRCA*1: 5 cases, *BRCA*2: 7 cases), and all patients had HGSC [12]. Furthermore, Hirasawa et al. analyzed 230 cases and reported 27 patients (11.8%) with g*BRCA*m; 19 patients (8.3%) in *BRCA*1; and 8 patients (3.5%) in *BRCA*2. By histological subtype of all 27 patients, 22 (81.5%) had HGSCs, and 2 patients (7.4%) had endometrioid carcinomas or clear cell carcinomas [13]. These results indicate the high frequency of clear cell carcinoma in Japan, with g*BRCA*m observed in 12% of all epithelial ovarian cancer cases, and no pathogenic variants in mucinous carcinomas. A similar trend was observed in Western countries. Recently, Enomoto et al. determined g*BRCA*1/2 pathogenic variants of Japanese patients in the Japan CHARLOTTE study [14]. The prevalence of g*BRCA* pathogenic variants by the FIGO stage was demonstrated in Figure 1. In stages I–II and stages III–IV ovarian cancer, the g*BRCA* pathogenic variant rate was 4.9% and 24.1%, respectively. Understanding the g*BRCA* pathogenic variant rate for each stage is very important for the explanation of companion diagnosis for PARPi use. The prevalence of g*BRCA*m by histological type is presented in Table 1. In stages I–IV, patients with high-grade serous, g*BRCA*m rate was 28.5%, low-grade serous was 20%, endometrioid was 6.7%, and clear-cell was 2.1%. On the other hand, in mucinous and seromucinous ovarian cancer, the prevalence was 0%. There are divisions of opinion regarding whether or not mucinous type is included in applicable patients of the companion diagnostics.

The prevalence of g*BRCA* pathogenic variant rate was different in each country. In patients with high-grade serous, the prevalence in Japan and China was higher than that of the U.S. [2,14,15]. In clear-cell carcinoma, the prevalence in Japan (2.1%) was lower than that of China and the U.S. Among epithelial ovarian cancers in Japan, the frequency of clear cell carcinoma is about 24%, which is about four times that in the USA. The reason is that the incidence of endometriosis is high in East Asia, especially in Japan [16].

**VUS:** variant of uncertain significance

### 2.3. Deciding Whether to Recommend gBRCA Test for All Ovarian Cancer Patients

As a companion diagnosis for PARPi use, there are ongoing discussions on whether or not *BRCA* genetic tests should be recommended for all ovarian cancer patients, whether there is the need for tests that consider family history and histological type, what time diagnostics should consider the use of PARPi, and how best to combine them with the analyses of surgical samples. As a genetic high-risk assessment for suspected HBOC families, there is a debate about the need for tests that consider family history and/or past history of HBOC related cancers, and the age of onset of ovarian cancer. Over 25% of *BRCA* pathogenic variant carriers develop ovarian cancer over the age of 60, and the age of onset of ovarian cancer does not correlate with the presence of g*BRCA* pathogenic variant [17,18,19,20,21]. The frequency of g*BRCA*m ovarian cancer patients without a family history is reported to be high in Western countries (35 to 40%) [17,18,19,20,21]. Likewise, in Japan, Sakamoto et al. observed that 17% (6/36) of patients with a family history and 10% (6/59) of patients with no family history had a g*BRCA*m, indicating that patients with no family history also had a comparatively high pathogenic variant frequency [12]. These data show the validity of recommending BRCA genetic testing for all ovarian cancer cases, regardless of family history. The reason for this recommendation can also be due to the effect of the degree of accuracy of family history hearings varying significantly between facilities. Subtle differences exist in the guidelines governing the recommendation of BRCA genetic testing for patients with suspected hereditary ovarian cancer in different countries. The guidelines of the National Comprehensive Cancer Network (NCCN) [22], the Society of Gynecologic Oncology (SGO) [23], and the American College of Obstetricians and Gynecologists (ACOG) [24] advocate for the consideration of BRCA genetic testing for ovarian cancer patients regardless of family history. On the other hand, the guidelines set by the European Society for Medical Oncology (ESMO), France, Germany, the Netherlands, Spain, and the UK (The National Institute for Health and Care Excellence; NICE) stated that genetic testing should be considered based on the presence/absence of family history of breast/ovarian cancer [25,26,27,28]. In addition, the guidelines of the Scottish Intercollegiate Guidelines Network (SIGN) stated that for nonmucinous ovarian cancer and fallopian tube cancer patients, genetic tests should be considered regardless of family history; they also recommended that the tissue type be kept in mind [29]. The BRCA test needs to be offered at the time of initial diagnosis to all patients with nonmucinous and nonborderline ovarian epithelial carcinoma, fallopian tube carcinoma, and primary peritoneal carcinoma in Scottish and Italian guidelines [30,31].

### 2.4. Correlation between HRD and Platinum/PARPi Sensitivity

PARP is an enzyme involved in DNA single-strand breaks (SSBs). SSBs are repaired via base excision repair, and double-strand breaks (DSBs) are repaired via homologous recombination (HR) [32,33,34]. In cells where SSBs are not repaired, DSBs occur when DNA is replicated. *BRCA* genes are involved in homologous recombination. Therefore, inhibiting PARP in cells with malfunctioning *BRCA* genes results in faulty DSB repair, failure of two mechanisms of DNA repair, and apoptosis being induced (synthetic lethality) [35].

**Figure 2:**
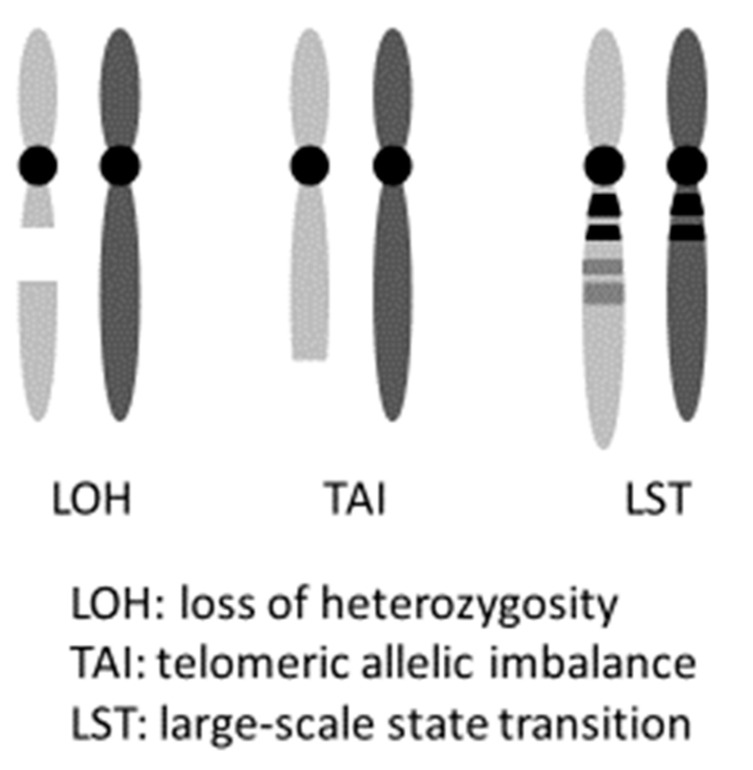
myChoice HRD score (Genomic instability status).

PARPi hold promise not only for induced synthetic lethality in HBOC ovarian cancers with a g*BRCA*m but also in tumors with HRD, including somatic *BRCA* pathogenic variant (s*BRCA*m). The term “*BRCA*ness” indicates HRD is caused by abnormalities that are not in the *BRCA* genes, but ultimately results in a state similar to having a *BRCA* pathogenic variant. It is possible to induce synthetic lethality in such cases using PARPi, as stated above. Clinically, cases that exhibit platinum-sensitivity often reflect this HRD status [36]. Hence, platinum sensitivity is used as a marker for PARPi effectiveness. Platinum causes the formation of cross linkages between complementary DNA strands. Hence, during repair, when DNA replication begins, the damaged regions result in a chain break, and homologous recombination is necessary. If homologous recombination occurs, the possibility of cell survival is increased. However, if homologous recombination fails, the cell loses its ability to repair the damaged DNA, resulting in cell death. As a result, platinum-sensitive ovarian cancer often has a deficiency in the homologous recombination repair mechanism (an example of HRD), proving the effectiveness of PARPi in this cancer. In practice, the frequency of HRD in cases of ovarian cancer indicates that while approximately 50% of HGSC cases have pathogenic variants in homologous recombination-related genes, HRD-related gene pathogenic variants are observed in 28% of nonserous cancers. This suggests that PARPi are effective in nonserous cases [37]. Analyses of 1915 cases of advanced ovarian cancer registered in two clinical trials (GOG 218 and GOG 262) indicated the presence of 14 homologous recombination-related genes (*BRIP1*, *PALB2*, *RAD51C*, *RAD51D*, *ATM*, *ATR*, *NBN*, *SLX4*, *BARD1*, *BLM*, *CHEK2*, *RBBP8*, *MRE11A*, and *XRCC2*), in addition to *BRCA1/2*. Patients with germline pathogenic variants in any of these 14 genes have roughly identical prognoses to patients with *BRCA*1 pathogenic variants. Both prognoses are more favorable than that of patients with no pathogenic variants in HR-related genes [2]. It has also been reported that loss of heterozygosity (LOH), telomeric allelic imbalance (TAI), and large-scale state transition (LST) can be effectively used as markers to evaluate a patient’s HRD score detected by Myriad myChoice^®^ HRD test [30,38]. Telomeric allelic imbalance (TAI) and large-scale state transition (LST) are defined respectively as “the large allelic imbalances extending into a telomere” and “the number of chromosomal breaks between adjacent regions of at least 10 Mb of differing allelic states” (Figure 2) [39,40]. This score system was used in the NOVA trial, which is a randomized phase III clinical trial of niraparib. A comparison of the niraparib group and a placebo group showed that the median value for progression-free survival (PFS) was significantly increased for the g*BRCA*m cohort (hazard ratio, 0.27; *p* <0.0001), as well as for the germline *BRCA* pathogenic variant-negative (g*BRCA*wt) and HRD-positive cohort (hazard ratio, 0.38; *p* <0.0001), and the total g*BRCA*wt cohort (hazard ratio, 0.45; *p* <0.0001) [9]. Furthermore, the ARIEL3 trial of Rucaparib also showed PARPi to be effective regardless of the *BRCA* and HRD status in patients with platinum-sensitive, recurrent, serous/endometrioid adenocarcinoma [41].

The loss of heterozygosity (LOH), telomeric allelic imbalance (TAI), and large-scale state transition (LST) can be effectively used as markers to evaluate a patient’s HRD score.

### 2.5. Deciding Whether to Perform gBRCA or HRD Test First

When considered as a companion diagnostic for PARPi, which test should be performed first depends on whether olaparib or niraparib is used. The g*BRCA* test is a germline test that interprets the results differently than the HRD test, which examines somatic changes in tumors. As mentioned above, many genes related to HRD have been reported, and the HRD test is considered to be a more comprehensive test in terms of examining the susceptibility of PARPi. However, the HRD test cannot determine whether the ovarian cancer patient is hereditary or not, so an additional g*BRCA* test is needed. It should be noted that s*BRCA*m accounts for 4–9% of tumor *BRCA* pathogenic variant (tBRCAm), and 90% or more of t*BRCA*m is considered to be g*BRCA*m [42]. It is important to discuss whether or when to perform the HRD test or the gBRCAm test. The benefits of using g*BRCA*m are that it can (1) predict super-responders for PARPi, (2) select fertility preservation surgery in young ovarian cancer patients, (3) contribute to the prevention of secondary cancer in the patient, and (4) evaluate the detailed cancer risk in the family member.

The myChoice^®^ HRD test is used as a companion diagnostic for treatment decisions in maintenance therapy after initial chemotherapy (olaparib) and treatment decisions for recurrent cancer with a history of 3 or more regimens (niraparib). The HRD test *is used as *HRD* and tBRCAm test*. It is based on the results of the SOLO1, PAOLA-1, and QUADRA trials [7,8,43]. Two problems of the HRD test are found thus far: (1) the assay method has not been established and (2) whether the recurrent tumor reflects the characteristics of the tumor removed in primary surgery. Regarding the judgment result of the HRD test, the cutoff value differs depending on the clinical trial in PRIMA, NOVA, AVANOVA2, PAOLA-1, QUADRA trial (cutoff point = 42), and VELIA trial (cutoff point = 33), respectively [7,8,9,10,43,44]. The HRD score is calculated by summing three independent parameters: LOH, TAI, and LST in the tumor [9,30,38,39,40,45]. A HRD score ≥42 has been shown to predict the susceptibility to PARPi therapy independent of *BRCA* pathogenic variants [8,43]. Takaya et al. reported that *BRCA* pathogenic variants were enriched in the group with HRD scores ≥63 in high-grade serous ovarian carcinoma, and this group had a good prognosis [46]. How et al. reported that a HRD score ≥33 was associated with improved overall survival in ovarian cancer [47]. Since the HRD test is a somatic genetic test, there is concern that some doctors can easily perform the test. Such an attending physician may not provide adequate genetic counseling and the g*BRCA*m test, after the t*BRCA*m results are known. Given that situation, if heredity is suspected due to past medical history, family history and/or histological type of ovarian cancer, it may be better to perform the gBRCA test first for the patient and her family. The HRD test is not required as a companion diagnostic in patients who use niraparib as maintenance therapy after initial treatment. However, in the PRIMA, SOLO1, and PAOLA-1 trials, PARPi have been reported to be most effective in patients with g*BRCA*m [7,8,10]. Therefore, it is necessary to determine which test should be performed first in each patient based on family history, past medical history, histology of ovarian cancer, and treatment policy.

BRACAnalysis^®^ CDx is used as a gBRCA test; on the other hand, myChoice® HRD test* and* FoundationOne^®^CDx is used as tBRCAm and HRD test. In the positive case of t***BRCA***m or HRD test, germline single-site testing needs to be considered as a definitive diagnosis of hereditary tumors. However, the HRD test using FoundationOne^®^ CDx or myChoice^®^ HRD can provide information on many HR-related genes, making it a more useful test for examining PARPi sensitivity. Heeke et al. reported that molecular profiles of 52,426 tumors were reviewed to identify pathogenic variants in the HR involved genes, *ARID1A*, *ATM*, *ATRX*, *BAP1*, *BARD1*, *BLM*, *BRCA1/2*, *BRIP1*, *CHEK1/2*, *FANCA/C/D2/E/F/G/L*, *MRE11A*, *NBN*, *PALB2*, *RAD50*, *RAD51*, *RAD51B*, or *WRN* [48]. As a result, the overall frequency of the pathogenic variants detected was 17.4%, while endometrial was 34.4% (*n* = 1475) and ovarian was 20.0% (*n* = 2489).

## 3. Risk-Reducing Salpingo-Oophorectomy for BRCA Pathogenic Variant Carriers

The greatest advantage of *BRCA* genetic testing for people with relatives with a history of HBOC is the option of risk-reduction surgery. The NCCN guidelines recommended that *BRCA* pathogenic variant carriers have a RRSO if they are 35 to 40 years old [22]. If there have been cases of ovarian cancer at younger ages in the family, RRSO may be considered by that age. For women who do not opt for RRSO, surveillance with a combination of transvaginal ultrasounds and CA125 tests may be done at the discretion of the attending physician, although it was clearly demonstrated that the benefit of the surveillance has not been established. It can be gathered that there is an emphasis on the effects of RRSO and breast cancer risk-reduction.

A meta-analysis of the breast/ovarian cancer risk-reducing effect of RRSO showed that ovarian cancer risk was reduced by 79% and breast cancer risk by 51% [49]. Removing premenopausal ovaries holds promise for reducing the risk of developing estrogen-dependent breast cancer, but hereditary breast cancer is often triple-negative, so it may also be caused by nonhormonal mechanisms. Although it is almost certain that the risk of ovarian cancer is most likely reduced, Heemskerk-Gerritsen et al. reported that RRSO did not reduce the risk of breast cancer for *BRCA* pathogenic variant carriers [50]. In addition, a prospective multicenter cohort study reported that RRSO significantly reduced the risk of breast cancer for *BRCA*2 pathogenic variant carriers but not for *BRCA*1 carriers [51]. Further studies are to be conducted on the effect of reducing the risk of breast cancer. A prospective cohort study of 2482 *BRCA* pathogenic variant-positive people by Domcheck et al. and the meta-analysis by Marchetti et al. showed that RRSO reduced all-cause mortality by 60% and 68% respectively, in both pre- and postmenopausal women [52,53]. Table 2 summarizes the details of the risk reduction effect of RRSO against ovarian and breast cancer.

A prospective trial has begun on prophylactic salpingectomy with delayed oophorectomy (PSDO) in women who had their ovaries removed at age 40 or 50 [54,55]. However, it has been reported that only 40 to 60% of HGSC develop in fallopian tubes [56,57]. Hence, the NCCN guidelines stated that the protective effect of salpingectomy alone has not been demonstrated, and it is not a standard risk-reduction treatment. In addition, salpingectomy alone was said to not decrease the risk of developing ovarian or breast cancer, so further discussion is evidently required. There is still no consensus on whether to remove the uterus when performing RRSO. One report stated that there is no clear increase in the risk of endometrial cancer after RRSO, while another report noted that the risk of serous endometrial carcinoma was increased in *BRCA*1 carriers [58]. Hysterectomy may be beneficial when hormone replacement therapy is needed for the patients undergoing RRSO [59,60].

Current evidence does not strongly recommend hormone replacement therapy (HRT) for gBRCAm carriers with a history of breast cancer, but if the hormone receptor is negative, HRT may be considered. On the other hand, there is no strong evidence that HRT increases the incidence of breast cancer, ovarian cancer, and endometrial cancer for those without a history of breast cancer, so it is acceptable to perform HRT to reduce the symptoms associated with menopause [61,62]. However, the risk of developing breast cancer by HRT may differ between *BRCA1* and *BRCA2* pathogenic variant carriers, and therefore we need to continue discussing carefully whether long-term estrogen treatment does not really increase the risk of breast cancer incidence.

## 4. Conclusions

In the field of gynecological oncology, there are still few molecular target drugs that can be used in comparison to other areas; however, the approval of the PARPi has been changing the treatment of ovarian cancer patients at present. In combination with bevacizumab and the use of niraparib, HRD tests in tumors have begun to be performed in clinical practice, and ovarian cancer treatment is changing rapidly. Regarding RRSO, it is necessary to pay attention to results of ongoing studies, such as the appropriate age to undergo surgery, the combined resection of the uterus, and risk-reducing salpingectomy. Future discussions are also needed on personalized medical care due to differences in mutant genes and genotypes.

## Figures and Tables

**Table 1:** Prevalence of g*BRCA*m by histological type. NA: not applicable.

Histological Classification	USA [2] (*n* = 1915)	China [15] (*n* = 1331)	Japan [14] (*n* = 634)
High-grade serous	16.0%	27.2%	28.5%
Mucinous	0.0%	7.0%	0%
Endometrioid	10.9%	10.8%	6.7%
Clear cell	6.9%	7.6%	2.1%
Seromucinous	NA	NA	0%

**Table 2:** Risk reduction effect of RRSO against ovarian cancer and breast cancer.

Author	Year	Study Design	Ovarian Cancer HR (95% CI)	Breast Cancer HR (95% CI)	Total Mortality HR (95% CI)
Rebbeck et al. [49]	2009	Meta-analysis	0.21 (0.12–0.39)	0.49 (0.37–0.65)	NA
Marchetti et al. [53]	2014	Meta-analysis	0.19 (0.13–0.27)	NA	NA
Domchek et al. [52]	2010	Prospective cohort	0.28 (0.12–0.69) *	0.54 (0.37–0.79) *	0.40 (0.26–0.61)
			0.14 (0.04–0.59) †	1.00 (0.56–1.77) †	
Kauff et al. [51]	2008	Prospective cohort	0.15 (0.04–0.56) ‡	0.61 (0.30–1.22) ‡	NA
				0.28 (0.08–0.92) §	

* with no breast cancer history, NA: not applicable, † with breast cancer history, ‡ BRCA1, § BRCA2. HR: Hazard Ratio; CI: confidence interval

## Data Availability

Not applicable.
